# Author Correction: Signatures of Mottness and Hundness in archetypal correlated metals

**DOI:** 10.1038/s41467-020-14420-y

**Published:** 2020-01-21

**Authors:** Xiaoyu Deng, Katharina M. Stadler, Kristjan Haule, Andreas Weichselbaum, Jan von Delft, Gabriel Kotliar

**Affiliations:** 1 Department of Physics and Astronomy, Rutgers University, Piscataway, NJ 08854 USA; 2 Physics Department, Arnold Sommerfeld Center for Theoretical Physics and Center for NanoScience, Ludwig-Maximilians-Universitat München, 80333 München, Germany; 3 Condensed Matter Physics and Materials Science Department, Brookhaven National Laboratory, Upton, NY 11973 USA

**Keywords:** Electronic structure, Electronic properties and materials, Theoretical physics

Correction to: *Nature Communications* 10.1038/s41467-019-10257-2, published online 20 June 2019.

The original version of this Article contained an error in the Acknowledgement. The correct Acknowledgement should have read:

Work by X.D. and G.K. was supported by NSF DMR-1733071. Work by K.H. was supported by NSF DMR 1405303. K.M.S. and J.v.D. acknowledge support from the excellence initiative NIM; A. W. was supported by the U.S. Department of Energy, Office of Basic Energy Sciences, under Contract No. DE-SC0012704.

This has now been corrected in both the PDF and HTML versions of the Article.

