# Supplementary material for: Signatures of Mottness and Hundness in archetypal correlated metals
Source: Nat Commun. 2019 Jun 20;10:2721. doi: 10.1038/s41467-019-10257-2 (PMC6586627; doi:10.1038/s41467-019-10257-2)
Supplement: Supplementary file 1 — Supplementary Information [file 41467_2019_10257_MOESM1_ESM.pdf]

Signatures of Mottness and Hundness in archetypal correlated metals: Supplementary Information

Xiaoyu Deng,[1,*] Katharina M. Stadler,[2,*] Kristjan Haule,[1]
Andreas Weichselbaum,[3,2] Jan von Delft,[2] and Gabriel Kotliar[1,3]

[1] *Department of Physics and Astronomy, Rutgers University, Piscataway, New Jersey 08854, USA*
[2] *Physics Department, Arnold Sommerfeld Center for Theoretical Physics and Center for NanoScience, Ludwig-Maximilians-Universitat München, 80333 München, Germany*
[3] *Condensed Matter Physics and Materials Science Department, Brookhaven National Laboratory, Upton, New York 11973, USA*

(Dated: April 4, 2019)

* These authors contributed equally to this work. Correspondence: xiaoyu.deng@gmail.com.

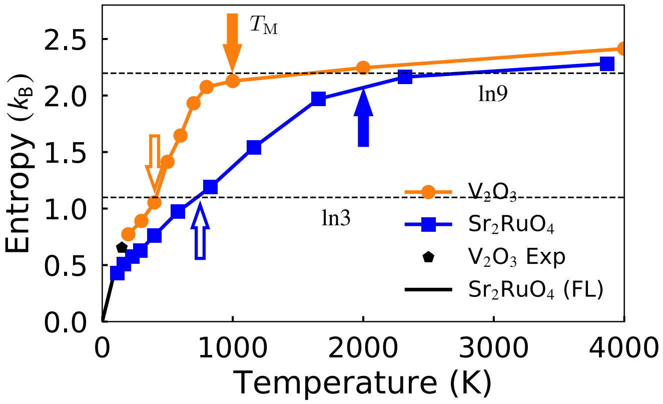

Supplementary Figure 1. The impurity entropy per V/Ru atom computed with DFT+DMFT in V_2O_3 (orange) and Sr_2RuO_4 (blue). A plateau at about ln(9) is seen in both materials, starting approximately at 1000K in V_2O_3 and approximately at 2000K in Sr_2RuO_4 (filled arrows). As temperature decreases, the entropy crosses ln(3) continuously, at about 400K in V_2O_3 and about 750K in Sr_2RuO_4 (open arrows). The black pentagon denotes an estimation of the entropy in V_2O_3 from experimental measurements [4–6]. The black line indicates a Fermi-liquid approximation of the electronic entropy of Sr_2RuO_4, $S = \gamma T$, taking the specific heat coefficient $\gamma = 38\text{mJmol}^{-1}\text{K}^{-2}$ at low T [14].

Supplementary Note 1: Temperature Evolution of Impurity Entropy in V_2O_3 and Sr_2RuO_4

We have computed the entropy of the correlated atom in both V_2O_3 and Sr_2RuO_4 up to high temperature within the DFT+DMFT framework. The results are depicted in Supplementary Figure 1. Notably in both systems, the impurity entropy initially shows a plateau at high temperature. As the temperature decreases, the entropy decreases markedly below about $T_M = 1000$K in V_2O_3 and below $T = 2000$K in Sr_2RuO_4. The value of the plateau is approximately ln(9) for both materials. This indicates that both spin and orbital degrees of freedom contribute significantly to the entropy in both materials and the most relevant atomic states likely have large spin $S = 1$ and large effective orbital angular momentum $L = 1$, as discussed in models [1, 2].

It is not surprising that in V_2O_3 the entropy plateau holds down to the same characteristic temperature $T_M = 1000$K, where the Curie behavior in the spin and orbital susceptibility ceases to exist, since the spin and orbital degrees of freedom remain free with decreasing temperature down to T_M. In Sr_2RuO_4 the entropy departs from the plateau at much larger temperature than in V_2O_3 and the characteristic temperature $T = 2000$K is close to the temperature at which the Curie behavior ceases in the spin susceptibility $T_{\text{spin}}^{\text{onset}}$. We note that at this temperature the orbital degrees of freedom might already be close to fully delocalized although even the highest temperatures studied here are too low for the orbital susceptibility to exhibit a Curie behavior, as shown by the $T\chi_{\text{orb}}(0)$ (Main Text, Fig. 3(c)). This is supported by the observation that, at around $T = 2000$K, the slope of $T\chi_{\text{orb}}(0)$ increases significantly with decreasing temperature. Therefore, the entropy contribution due to orbital degrees of freedom is large. In addition, the large charge fluctuation in Sr_2RuO_4 may contribute to the entropy accumulation as well. As temperature decreases, the entropy decreases continuously and crosses a value ln(3) expected for an unscreened $S = 1$ atomic state. We note that the crossing occurs at a temperature when the orbital degrees of freedom are (almost) fully screened: in V_2O_3 at about 400K, and in Sr_2RuO_4 at about 750K (Supplementary Figure 1). Indeed, both temperatures are roughly comparable to the screening scale $T_{\text{orb}}^{\text{cmp}}$ in the corresponding materials. This is consistent with the observation that the orbital degrees of freedom are fully screened at much higher temperatures than the spin degrees of freedom in both materials. The spin degrees of freedom are responsible for the large entropy found in an extended temperature regime where the orbital degrees of freedom are frozen. Overall, these results suggest a strong correlation between the entropy accumulation and the unscreening of spin and orbital degrees of freedom.

Both V_2O_3 and Sr_2RuO_4 have a large entropy at the lowest temperature considered, which highlights the strong correlated nature of these two materials. Interestingly, the large values of entropy are found in experimental measurements. In V_2O_3 the entropy change across the transition from a metallic state to the antiferromagnetic state at $T = 150$K is as large as $0.65k_B$ [4–6]. Assuming that the electronic entropy of the ordered state is zero [7], this value provides an estimation of the entropy in metallic V_2O_3 and fits very well in our computed impurity entropy (Supplementary Figure 1). In Sr_2RuO_4, as a first order approximation, the electronic entropy of Sr_2RuO_4 can be written as $S = \gamma T$ where γ is the specific heat coefficient in the Fermi-liquid regime. The approximated entropy matches our computed result at about 100K. These agreements suggest that the large entropy in both materials are mainly due to the local correlated electrons. In connection with the evolution of the local spectra, we see that in a

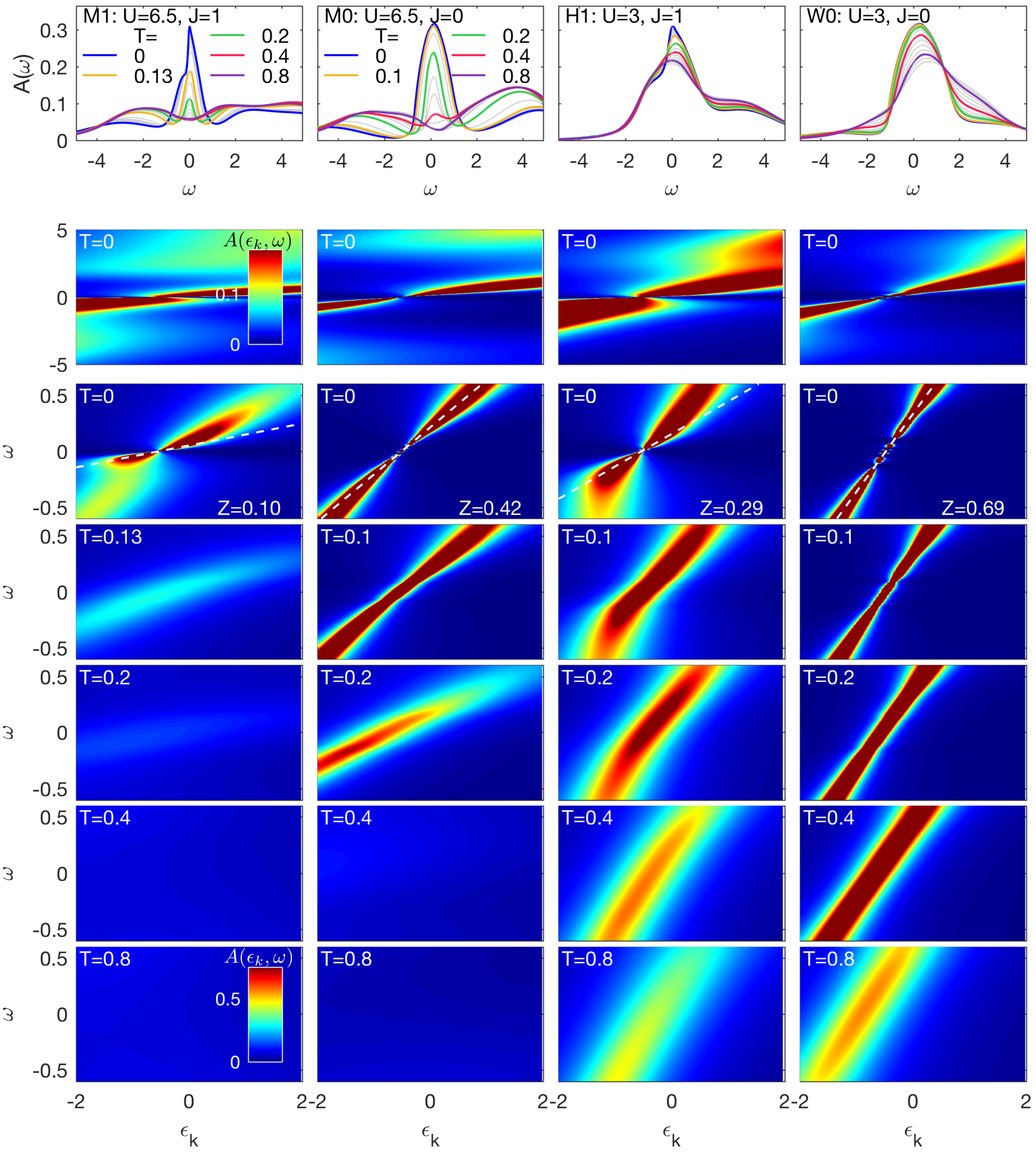

Supplementary Figure 2. Spectral data obtained for the 3HHM. Four parameter combinations marked by asterisks in Fig. 5(a) of the main text are considered: two Mott systems with $U = 6.5$ close to the Mott transition, having $J = 1$ (M1, first column) or $J = 0$ (M0, second column); and two systems with $U = 3$ far from the Mott transition, a Hund system with $J = 1$ (H1, third column) and a weakly correlated system with $J = 0$ (W0, fourth column). First row: local density of states $A(\omega)$ for various temperatures (the legend for M0 also applies to H1, W0). Second row: corresponding photoemission spectra $A(\epsilon_k, \omega) = -\frac{1}{\pi}\mathrm{Im}[\omega + \mu - \epsilon_k - \Sigma(\omega)]^{-1}$, computed using the standard DMFT protocol [20], at $T = 0$. Subsequent rows: low-frequency zooms of photoemission spectra, for five different temperatures.

large temperature range beyond the Fermi-liquid scale the large entropy is accompanied by a coherence resonance, a feature of resilient quasiparticles [8].

We remark that the large total orbital angular momentum $L = 1$ highlights the importance of the orbital degrees of freedom in the electronic structure. Its role has been emphasized in Sr_2RuO_4 [9, 10], however it is not much discussed in V_2O_3. The large orbital angular momentum in V_2O_3 is a direct consequence of the fact that the a_{1g} orbital is partially filled and contributes to the atomic degrees of freedom [11–13]. It is therefore very unlikely that the a_{1g} is

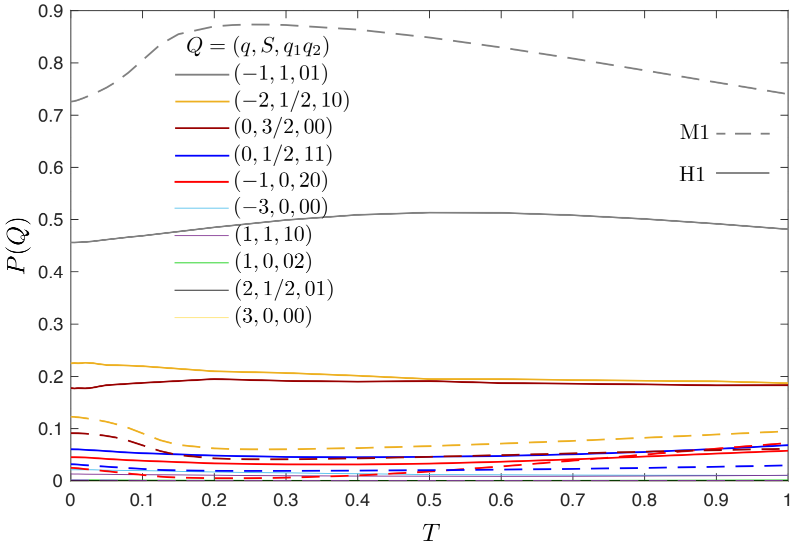

Supplementary Figure 3. Occupation probability of all energetically accessible atomic eigenstates as functions of temperature for M1 (dashed) and H1(solid). Eigenstates are labeled by their $U(1)_{charge} \times SU(2)_{spin} \times SU(3)_{orb}$ quantum numbers $Q = (q, S, q_1q_2)$ (see text).

effectively excluded by a correlation-enhanced crystal field splitting, as suggested by several studies [15–18]. This is consistent with the conclusion drawn in a recent angular-resolved photoemission spectroscopy measurement [19]. Our findings shed light on the nature of the Mott transition in V_2O_3.

Supplementary Note 2: Photoemission spectra for the 3HHM

To further elucidate the difference between Mott and Hund systems, Supplementary Figure 2 shows local spectra (first row) and photoemission spectra (subsequent rows) obtained from 3HHM calculations for four parameter combinations (described in the figure caption). We will discuss (i) the difference between large and small U, (ii) the difference between finite and zero J, and (iii) how J affects the slope of the dispersion in the photoemission plots.

(i) For the large-U Mott systems M1 and M0 in the first and second columns, the local spectrum $A(\omega)$ (first row) shows well-defined Hubbard side bands. At low temperatures these are separated by a central quasiparticle peak, which is suppressed with increasing T, giving way to a pseudogap. Correspondingly, in the photoemission spectra (subsequent rows) the spectral weight at low frequencies decreases very strongly as the temperature is increased. By contrast, for the Hund system H1 and weak Mott system W0 in the third and fourth columns, both having moderate U, the local spectra (first row) show no well-separated Hubbard side bands, and with increasing temperature the central quasiparticle peak weakens somewhat but persists, so that no pseudogap develops. Correspondingly, the photoemission spectra retain significant low-frequency weight even at high temperatures (subsequent rows).

(ii) For the finite-J systems M1 and H1 in the first and third columns, the lowest-temperature quasiparticle peak features a rather sharp, asymmetric central peak with a shoulder on its left flank. This substructure reflects J-induced spin-orbital separation, $T_K^{spin} \ll T_K^{orb}$, leading to a sharp Kondo resonance in the spin sector together with a broader Kondo resonance in the orbital sector [2, 3]. Correspondingly, this substructure is absent for the $J = 0$ systems M0 and W0 in the second and fourth columns.

(iii) The strength of the local correlations can be gauged from the slope of the low-energy dispersion relation, governed by the quasiparticle weight, $Z = m/m^*$. This quantity is proportional to the weight of the spin Kondo resonance [3]. Since finite J strongly reduces the latter, the low-temperature dispersion (for given U) has much smaller slope for J finite than zero (compare first to second column, and third to fourth). The fairly small slope of the dispersion for H1 (third column) compared to W0 (fourth column) thus is a smoking gun difference between a true Hund system and a pure Mott system tuned far from the Mott transition – even though both have rather weak U, the former has strong correlations (induced by finite J), the latter does not.

Supplementary Note 3: Mixed-valence effects in the 3HHM

In the 3HHM $T\chi_{orb}$ and $T\chi_{spin}$ follow Curie behavior (plateaus) for temperatures well above T_{orb}^{onset} or T_{spin}^{onset} (Main Text, Fig. 6(a,c)). However the plateau heights observed for M1 and H1 are only roughly comparable to the expected high-temperature asymptotics [21] for free local moments with given occupancy and spin configuration. In the following we demonstrate that these residual deviations can be ascribed to a mixed-valence regime at high temperatures.

Supplementary Figure 3 shows the occupation probability of all energetically accessible atomic eigenstates for M1 (dashed) and H1 (solid) as functions of temperature, where the collective quantum number $Q = (q, S, q_1 q_2)$ specifies the U(1) charge q, SU(2) spin S and SU(3) orbital representation $(q_1 q_2)$, labeled by a Young diagram with $q_1 + q_2$ (q_2) boxes in its first (second) row. For both systems, the state $Q = (-1, 1, 01)$ (grey), with charge $q = -1$ (one below half filling), spin 1 and orbital degree of freedom in the fundamental SU(3) representation, has by far the largest occupancy. For M1 (dashed grey) it dominates completely, with an occupation probability of almost 0.9 at $T \approx 0.2$, where $T\chi_{\mathrm{spin}}$ almost reaches 2/3 in Fig. 6(a) (of Main Text). At higher temperatures, other states become more important and the probability of $Q = (-1, 1, 01)$ decreases towards 0.7. By contrast, for H1, the occupancy of the state $Q = (-1, 1, 01)$ is around 0.5 (solid grey), and states with other charges, $q = -2$ (orange) and $q = 0$ (dark red), have appreciable occupancies, too. This indicates that mixed-valence effects are not entirely negligible.

[1] Georges, A., de'Medici, L. & Mravlje, J. Strong correlations from Hund's coupling. *Annual Review of Condensed Matter Physics* **4**, 137–178 (2013).
[2] Stadler, K. M., Yin, Z. P., von Delft, J., Kotliar, G. & Weichselbaum, A. Dynamical mean-field theory plus numerical renormalization-group study of spin-orbital separation in a three-band Hund metal. *Physical Review Letters* **115**, 136401 (2015).
[3] Stadler, K. M., Kotliar, G., Weichselbaum, A. & von Delft, J. Hundness versus Mottness in a three-band Hubbard-Hund model: on the origin of strong correlations in Hund metals. *Preprint at https://arxiv.org/abs/1808.09936* (2018).
[4] McWhan, D. B., Rice, T. M. & Remeika, J. P. Mott transition in Cr-doped V_2O_3. *Physical Review Letters* **23**, 1384–1387 (1969).
[5] McWhan, D. B., Menth, A., Remeika, J. P., Brinkman, W. F. & Rice, T. M. Metal-insulator transitions in pure and doped V_2O_3. *Physical Review B* **7**, 1920–1931 (1973).
[6] McWhan, D. B. *et al.* Heat capacity of vanadium oxides at low temperature. *Physical Review B* **7**, 326–332 (1973).
[7] A small entropy in the antiferromagnetic phase of V_2O_3 is expected since the paramagnetic-anfiferromagnetic transition is first-order and the atomic fluctuations are quenched by the long-range order. The estimated entropy is consistent with the estimation using the low temperature specific heat coefficient in the Fermi liquid state, as is done for Sr_2RuO_4 in the supplementary. The Fermi liquid state is reached by quenching the antiferromagnetism by doping or pressure and the corresponding specific heat coefficients in different samples are reported [4, 6].
[8] Deng, X. *et al.* How bad metals turn good: Spectroscopic signatures of resilient quasiparticles. *Physical Review Letters* **110**, 086401 (2013).
[9] Mravlje, J. *et al.* Coherence-incoherence crossover and the mass-renormalization puzzles in Sr_2RuO_4. *Physical Review Letters* **106**, 096401 (2011).
[10] Mravlje, J. & Georges, A. Thermopower and Entropy: Lessons from Sr_2RuO_4. *Physical Review Letters* **117**, 036401 (2016).
[11] Deng, X., Sternbach, A., Haule, K., Basov, D. N. & Kotliar, G. Shining light on transition-metal oxides: Unveiling the hidden fermi liquid. *Physical Review Letters* **113**, 246404 (2014).
[12] Held, K., McMahan, A. & Scalettar, R. Cerium volume collapse: Results from the merger of dynamical mean-field theory and local density approximation. *Physical Review Letters* **87** (2001).
[13] Leonov, I., Anisimov, V. I. & Vollhardt, D. Metal-insulator transition and lattice instability of paramagnetic V_2O_3. *Physical Review B* **91**, 195115 (2015).
[14] Mackenzie, A. P. *et al.* Observation of quantum oscillations in the electrical resistivity of $SrRuO_3$. *Physical Review B* **58**, R13318–R13321 (1998).
[15] Poteryaev, A. I. *et al.* Enhanced crystal-field splitting and orbital-selective coherence induced by strong correlations in V_2O_3. *Physical Review B* **76**, 085127 (2007).
[16] Hansmann, P. *et al.* Mott-Hubbard transition in V_2O_3 revisited. *physica status solidi (b)* **250**, 1251-1264 (2013).
[17] Grieger, D. & Lechermann, F. Effect of Chromium doping on the correlated electronic structure of V_2O_3. *Physical Review B* **90**, 115115 (2014).
[18] Grieger, D. & Fabrizio, M. Low-temperature magnetic ordering and structural distortions in vanadium sesquioxide V_2O_3. *Physical Review B* **92**, 075121 (2015).
[19] Lo Vecchio, I. *et al.* Fermi surface of metallic V_2O_3 from angle-resolved photoemission: Mid-level filling of e_g^π bands. *Physical Review Letters* **117**, 166401 (2016).
[20] Georges, A., Kotliar, G., Krauth, W. & Rozenberg, M. J. Dynamical mean-field theory of strongly correlated fermion systems and the limit of infinite dimensions. *Reviews of Modern Physics* **68**, 13 (1996).
[21] Hanl, M. and Weichselbaum, A. Local susceptibility and Kondo scaling in the presence of finite bandwidth. *Phys. Rev. B* **89**, 075130 (2014).
